# Marriage Intention among Korean Young Adults: Trends and Influencing Factors

**DOI:** 10.3390/ijerph19148557

**Published:** 2022-07-13

**Authors:** Doyeon An, Sang-Lim Lee, Hyekyung Woo

**Affiliations:** 1 Department of Health Administration, Kongju National University, Gongju 32588, Korea; chnsun2411@smail.kongju.ac.kr; 2 Korea Institute for Health and Social Affairs, Sejong 30147, Korea; dearlim@gmail.com; 3 Institute of Health and Environment, Kongju National University, Gongju 32588, Korea

**Keywords:** young adults, difference, trend, marriage, intentions

## Abstract

The recent increase in the tendency of people to marry late or to opt out of marriage entirely is among the key contributors to Korea’s low fertility rate. One possible cause of this tendency may be a change in how marriage is valued among Korea’s youth. The marriage intentions of young adults can be classified into “positive”, “negative”, and “neutral”. Over time, positive marriage intentions have declined across all age groups (2010: 61% → 2020: 39%; ages 25–29), with no significant change in negative marriage intentions. In contrast, neutral marriage intentions have increased significantly (2010: 36% → 2020: 53%; ages 25–29). This phenomenon may be attributable to the increase in the number of young adults who prioritize survival over thinking about the future. However, neutral marriage values can be changed into positive values at any time. A holistic overview of Korean society is necessary to determine how the values of young adults might be influenced to align with a traditional life process.

## 1. Introduction

Young adults’ tendency to marry late or to opt out of marriage entirely has been identified as a key factor in Korea’s low birth rate. In 2020, the average age at first marriage in Korea was 33.2 years for men and 30.8 years for women; this is expected to continue rising [1]. According to the results of the 2010 and 2020 Housing Population Census, the proportion of unmarried people in their early and late 30s increased over the 10-year interval to 37.8%. For women, it increased significantly from 29.1% to 50.2% in their early 30s and from 12.6% to 26.7% in their late 30s [1]. Most births in Korea occur after marriage; therefore, young adults’ decisions to marry late or not to marry at all have been identified as an aggravating factor in the declining fertility rate [2].

Social phenomena are not composed of a single isolated event, but rather they are caused by several complex events that may be economic, social, or cultural in nature [3]. This means that social phenomena are not taking place under a single condition alone. The causes of the increased numbers of unmarried people and those marrying late should also be investigated from this perspective. Young adults’ changing values, particularly with respect to marriage, are among the key factors that have contributed to this phenomenon [4,5], as recent changes in the younger generation’s values have directly impacted marriage rates [2,6]. Demographic factors, subjective values, and family environment contribute to the change in young people’s values. In this regard, academic interest in young adults’ backgrounds [7], subjective characteristics [8,9], and family characteristics [10,11,12] is increasing.

Today’s younger generation have a greater responsibility for the formation of their own values and prize individuality to a greater extent than any preceding generation [13,14]. The diversification of information access methods over time has facilitated increased contact with diverse cultures, allowing young adults greater freedom to develop more diverse value systems than the previous generation. However, society tends to regard individual generations as monolithic groups, and Korea’s younger generation has been classified into various cohorts, such as the “880,000 Won Generation” and the “Candlelight Generation” [15]. Therefore, diversity among the younger generation has thus far been neglected in scholarly discussion. While numerous discourses and studies have sought to identify the general characteristics of the younger generation [14,16,17,18,19], explorations of the differences and determinants that exist within that generation as well as temporal tendencies and cohort discriminatory characteristics as manifested in time series are relatively rare.

As noted, values and attitudes relating to marriage play a critical role in the implementation of marriage [3]. It is thus necessary to closely examine how the younger generation’s values have changed, along with the factors that influence such changes. Most studies on Korea’s low fertility rate to date have focused on younger people aged between their 20s and 40s as a single group [5,20,21,22,23]. In consideration of the diversity that exists and is evolving among the younger generation, a more nuanced approach is warranted. Accordingly, this study aimed to identify the younger generation’s demographic and social characteristics by investigating changes in young adults’ marriage intentions and identifying the factors that have led to these changes.

### 1.1. Concepts of and Influences on Marriage

Marriage marks the initiation of the family system and is a fundamental social institution, irrespective of time or cultural context [12,24,25]. The family is society’s smallest unit and plays a fundamental role in societal formation. In Korean society, which has a firmly established employment–marriage–childbirth life cycle, marriage has been essential to family formation [7].

Marriage is considered and opted into based on several factors and demographic characteristics such as gender, place of residence, education level, and economic activity influence decisions surrounding marriage [6,7,20]. In terms of gender, women tend to be more negative than men in their attitudes toward marriage. This is likely to be because women are expected to assume most of the responsibility for housework in addition to employment outside the home [10,26]. Economic activity also has an influence on attitudes toward marriage: men whose quality of employment is good are more likely to marry [16,22]. Differences in the environment according to the place of residence also affect marriage [5,17]. Educational level is also an economic resource for men and acts as an influencing factor on marriage. For men, the higher their education level, the more influence it will have on their attitude toward marriage [3,22,27], while women’s education shows no significant effect [22]. However, women find it more difficult than men to persist with their studies after marriage, and so the higher their level of education, the more likely they will be to delay marriage [7].

Marriage is influenced not only by these characteristics but also by family and subjective evaluation characteristics [11,28,29]. Family values have been shown to influence attitudes toward marriage [10], and implementation of marriage also changes according to personal values. These values further differ according to gender and group [3,5,10,20,29,30]. As intentions to marry vary from group to group, it is crucial to explore the role played by familial and subjective characteristics. Few detailed studies on groups within the younger generation have been published to date. Accordingly, it is necessary to understand to what extent the demographic characteristics such as gender, place of residence, education level, and economic activity, as well as characteristics such as family and subjective evaluation affect the marriage intentions of young adults.

### 1.2. Marriage Values and Intentions

Marriage values include an individual’s attitudes or perceptions that determine their choice of spouse or decision on whether to marry [29,31]. They may also be defined as the criteria or perspectives that determine how the family unit is valued [11,20], the degree of intention to marry, and the attitudes towards and perceptions of marriage. Attitudes toward and perceptions of marriage vary according to context and are influenced by individual, social, cultural, and economic factors [31,32]. These values are reflected by the increased age at first marriage, increasing numbers of single and non-married individuals, and changes in perceptions of marriage, childbirth rates, and the economy [33,34]. Marriage intention may be regarded as an individual’s likelihood of actually transitioning to marriage [11,31,35].

Marriage is undertaken based on individual desires and needs [29]. As social values have come to be considered more important than individuals’ desires, the degree of subjective satisfaction has become an important factor in marriage decisions [36]. Those who are subjectively satisfied with their future prospects or who have positive social awareness are more likely to regard marriage in a positive light [19]. In addition, changes in family-related values as well as subjective future prospects and social awareness are strongly related to the phenomenon of late marriage and non-marriage among single men and women [20]. Several previous studies have shown that young adults’ differing values affect the necessity of or attitudes toward marriage [34,37]. However, most studies investigated only fragmentary groups, such women or workers exclusively [26,28,29,38]. Considering the diversification of young adults’ values and cross-regional differences, it is necessary to adopt a more detailed and nuanced approach.

## 2. Materials and Methods

### 2.1. Analytical Data and Research Subjects

This study used social survey data from the National Statistical Office of Korea [39]. The social survey took a sample of 27,336 households from 1548 survey districts across the country and asked people over the age of 13 about their social concerns and subjective consciousness in relation to their quality of life. The survey yields cross-sectional nationwide data on an annual basis. The data are categorized into family, education and training, health, crime and safety, living environment and welfare, social participation, culture and leisure, income and consumption, and labor every two years. This study used data from 2010 to 2012, 2014, 2016, 2018, and 2020, when a survey on family and marriage views was conducted to assess young adults’ changing perceptions of marriage in detail.

The subjects consisted of unmarried men and women aged 20–35 years. From 2010 to 2020, a total of 44,780 men and women aged between 20 and 35 were surveyed. For accurate analysis, respondents who were married, divorced, or widowed and those who did not respond to the question regarding marriage intention or who answered “I do not know” were excluded from the analysis. A total of 42,593 respondents were ultimately included in the analysis, excluding all missing values among the variables used.

### 2.2. Variables

Youth has been defined differently in various studies and policies [5,15,38,40,41], and no clear definition has yet been established. Most studies have regarded youth as ranging from 20 to 44 years [5,20,21,22,23,38]. Another study using social survey methods defined youth as ranging from 25 to 39 years [5]. The present study applied the following classifications: 20–24 = early youth, 25–29 = middle youth, and 30–34 = late youth [19].

Marriage intention was selected as a dependent variable to assess changes in young adult’s values with respect to marriage. In the question concerning “intentions on marriage”, “You must do it” and “It’s better to do it” were classified as positive marriage intentions; “It is okay to do it; it is not necessary to do it” was classified as a neutral marriage intention; and “I prefer not to do it” and “I should not” were classified as negative intentions [2,42].

Demographic variables included gender, region of residence, and final educational attainment level [23,38]. Gender was classified into “male” and “female”; residential area was classified into “metropolitan area” and “non-metropolitan area”. Final educational attainment level was classified into “under high school”, “university (2–3 years)”, “university (4 years)”, and “graduate school”. Economic activity and household income were used as economic variables. Regardless of job search activity, economic activity was classified as “doing” if economically active, and “not doing” if not economically active. Household income was classified as: under 2 million won, 2–4 million won, 4–6 million won, and 6 million won or more. Subjective satisfaction, subjective health, and subjective stress were used as subjective variables [28]. For subjective satisfaction, “very satisfied” and “slightly satisfied” were classified as satisfactory. Subjective satisfaction is a question that determines how much satisfaction you feel in your daily life. For subjective health evaluation, “very good” and “good enough” were classified as healthy using “health evaluation” items. Subjective stress was classified into “feel very much” and “feel quite a bit” using the “stress level” question to measure daily stress levels. Family characteristic variables included family relationships and number of family members [10]. For family relationships, the overall family code was used in the “family relationship satisfaction” question. It comprised of “very satisfied” and “very dissatisfied” and was classified as good, average, or bad. The number of family members was classified into one, two, three, and four or more.

### 2.3. Analysis

In this study, the chi-square test was performed to analyze the frequency between the variables, and Bonferroni’s test was performed as a post hoc test. Using a Lexis diagram, we compared the change in marriage intentions according to the subject’s gender. In the Lexis diagram, the horizontal axis represents the period, the vertical axis represents age, and the diagonal line represents the cohort [43]. It facilitates easy and swift comparison of changes in population characteristics as all age–period cohorts can be displayed in one graph. To use the Lexis diagram, the period and age should have the same interval. In this study, the age interval was analyzed based on the same two-year unit according to the two-year investigation period.

To examine changes in the factors that influence marriage intention in diverse groups within the younger generation, logistic regression analysis was performed by classifying the groups as follows: 20 to 24, 25 to 29, and 30 to 34. To confirm the variables’ characteristics, descriptive statistics were presented for each marriage intention and the analysis was performed according to age group by dividing them into men and women. When examining the differences in the factors that influence men and women, each variable was first corrected by introducing the residential area, final academic background, subjective evaluation characteristics, and family characteristics. We subsequently compared the odds ratio of neutral and negative intentions with positive intentions. R version 4.0.3 and IBM SPSS Statistics (version 27.0) was used for statistical analysis.

## 3. Results

### 3.1. Demographic Characteristics of Youth by Marriage Intention

Table 1 presents the results of the comparison of the different youth groups’ marriage intentions and marriage intentions according to demographic characteristics. The results are as follows for positive marriage intention: 20–24 years = 29%, 25–29 years = 33%, and 30–34 years = 38%. The neutral marriage intention results were as follows: 20–24 years = 30%, 25–29 years = 31%, and 30–34 years = 39%. As such, the 30–34 age group accounted for the highest proportion of both neutral and positive marriage intentions. Regarding negative marriage intention, the 20–24 age group showed the highest proportion of the results, with 36%, followed by 33% for the 25–29 age group and 31% for the 30–34 years age group (*p* < 0.001). In terms of gender, males accounted for a slightly higher proportion of positive intentions, with 56.6%, while females accounted for a slightly higher proportion of neutral and negative intentions, with 59% and 66%, respectively (*p* < 0.001). With respect to residential area, non-metropolitan areas were more frequent than metropolitan areas, and the proportion of metropolitan areas increased slightly as negative marriage intentions increased (*p* < 0.05). In terms of final educational attainment, the proportion of those who had attended university for four years was highest among all marriage intentions (*p* < 0.001).

In terms of economic activity, the percent of economically active individuals was generally high but declined from positive through neutral to negative (*p* < 0.001). For household income, in the case of positive intentions, income of less than 2 million won accounted for 27%, 2–4 million won accounted for 44%, 4–6 million won accounted for 19%, and 6 million won or more accounted for 10% of responses. Both neutral and negative intentions showed similar rates to positive intentions (*p* < 0.01). In the case of subjective satisfaction, positive and neutral intentions both showed low rates of poor satisfaction, but the negative rate doubled to 30% (*p* < 0.001). In the subjective health evaluation, from positive to negative, the positive intention percentages decreased, and the percentages of neutral and negative gradually increased (*p* < 0.001). Regarding subjective stress, the majority of all marriage intentions showed good results (*p* < 0.001). Regarding family relationships, those with good family relations showed the highest positive marriage intention rates. The proportion of people with normal family relations and those with poor family relations showed higher neutral and negative intentions (*p* < 0.001). Regarding the number of family members among those who expressed positive marriage intentions, 13% had one, 18% had two, and 31% had three, and 38% had four or more. The higher the number of family members, the higher the positive marriage intention rate, with similar rates for both neutral and negative intentions (*p* < 0.01). As for the survey year, the positive rate was highest in 2010 and declined up to 2020. The percentages of neutral and negative intentions increased from 2010 to 2020 and, in the case of negative intentions, 2020 showed the highest percentages (*p* < 0.001).

### 3.2. Changes in Marriage Intention of Unmarried Youth

**Figure 1:**
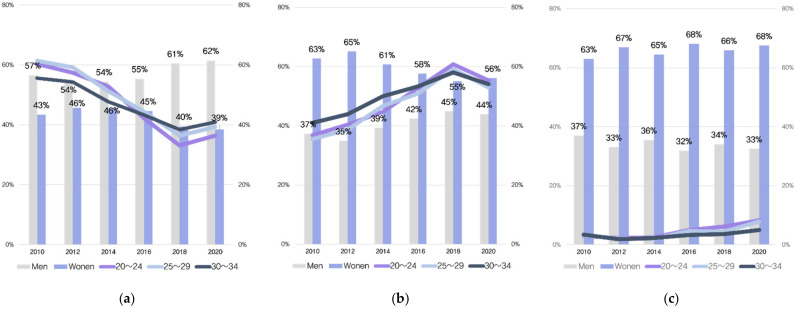
Changes in unmarried young adults’ (aged 20–34) intentions to marry by year: (**a**) positive marital intentions, (**b**) neutral marital intentions, and (**c**) negative marital intentions.

Figure 1 illustrates the marriage intention trends among Korea’s unmarried youth for the 10-year period from 2010 to 2020. Over the last 10 years, positive marriage intentions have declined among Korea’s unmarried youth as neutral marriage intentions have increased. Marriage intentions were divided into positive, neutral, and negative, and changes were identified. The graph charting positive marriage intention showed a gradual decline over the period from 2010 to 2018. The year 2010 showed the highest positivity rate and in 2018 the results were as follows: 20–24 years old = 33%; 25–29 years = 36%; and 30–34 years = 38%—the lowest of the 10-year period. The graph charting negative marriage intentions showed only a slight increase.

Neutral marriage intentions, by contrast, showed a clear upward curve. The curves of the positive and neutral marriage intention graphs contrast with one another, but the negative marriage intention graph showed no meaningful change. This means that the young adults who had formerly held positive marriage intentions are shifting toward a more neutral position. The curve of the graph for marriage intention according to age group was similar. No individual differences emerged with respect to age, but similar graphs were drawn for all ages.

In terms of gender, more men than women expressed positive intentions. Gender ratios were most similar among those with positive marital intentions between 2012 and 2014 (men 54%, women 46%), but the proportion of women with positive intentions decreased sharply between 2016 and 2020 (46% → 39%). The proportion of men with neutral marriage intentions has increased since 2014 (39% → 44%). Women’s negative marriage intentions were twice those of men (men 33%, women 66% in 2020). This may be because women’s social burden after marriage is perceived to be greater than that of men. The unmarried rate in Korea began to increase significantly during the 2000s, a period during which women’s level of education rose rapidly and women’s social advancement increased significantly [42]. As women’s social advancement continues to increase, women face the challenge of fulfilling their roles in society in addition to fulfilling familial norms. Women’s negative marriage values were created as a result of negative marriage intentions that had accumulated based on their direct and indirect experiences.

**Figure 2:**
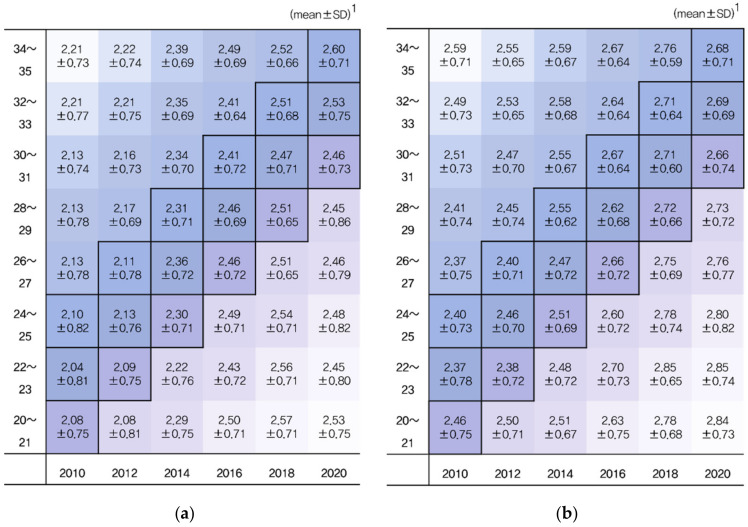
Marriage intention scores of unmarried young adults aged 20–35 according to gender: (**a**) changes in the marriage intention of men and (**b**) changes in the marriage intention of women. ^1^ Scoring for the “intention on marriage” question: 1 point “must do” to 5 points “should not”.

Analyses of the dynamic aspects of marriage intention among unmarried youth aged 20–35 according to gender using the Lexis diagram are as follows (Figure 2). In this study, the generation’s marriage intention score was measured using the mean and standard deviation by scoring the marriage intention questions from one to five, where one was very positive and five was very negative. Changes in marriage intention by year can be compared with the age of the same year based on the Lexis diagram’s vertical axis, while changes by year within the same age can be assessed based on the horizontal axis.

For marriage intention by generation, negative marriage intentions increased as the period progressed. Men’s marriage intention scores from 2010 to 2020 include men who were aged 20–21 in 2010 and 2012, 22–23 in 2014; 30–31 and 32–33 in 2016, 30–31 in 2018, and 22–23 and 28–29 in 2020. Women’s marriage intention scores include women who were aged 22–23 and 26–27 in 2010, 22–23 in 2012, 26–27 in 2014, 24–25 in 2016, 30–31 and 32–33 in 2018, and 30–31 in 2020. Men’s marriage intention scores were generally lower than those of women of the same age group. This may be because the burden on women after transitioning to marriage is greater than that on men.

Comparing the difference between values by year for the same age group, the average difference between 2010 and 2020 for men aged 20–21 was 0.45 points, and the average difference value for women was 0.38 points. The 10-year average difference value for men aged 34–35 was 0.39 points, and the average difference value for women was 0.09, which increased the marriage intention score of the younger group, particularly for men. All age groups increased their scores compared to the previous year. As of 2016, both women and men in the lower age group showed a significant change. The recent sharp decline in the number of people with positive marriage intentions indicates that traditional marital values are weakening.

Diagonals in the Lexis diagram represent cohorts. By observing a single point in time, the degree of change over time for a specific age can be considered from a lifetime perspective [43]. Both male and female birth cohorts had higher marital intention scores in 2020 than in 2010. From the cohort perspective, the overall score was higher for women, but the difference in scores according to the period was higher for men.

### 3.3. Marriage Intention of Unmarried Youth and Factors Affecting Marriage Intention by Characteristics

This analysis aimed to examine the factors that changed the marriage intentions of unmarried youth aged 20–34. Each variable was corrected by inputting the final educational background, subjective evaluation characteristics, and family characteristics variables. Classification according to group characteristics, such as gender and residential area, facilitated close examination of differences in factors affecting marriage intention between groups. Multinomial logistic regression analysis was performed to assess differences in attitudes and factors influencing marriage intention according to gender and residence type. The results of the analysis show the effect of the relative odds ratio belonging to the “neutral” and “negative” groups using the group with positive marriage intentions as the reference group for the dependent variable. The odds ratio of the independent variables represents the probability of belonging to this group based on the “positive” group—the reference group for the dependent variable—rather than the probability of belonging to the negative group.

The results of the multinomial logistic regression analysis for the positive determinants of marriage intention for unmarried men aged 20–34 are shown in Table 2 below. The lower the final educational level, the higher the probability of neutral or negative intentions among men aged in their 30s or older, more so than for men in their 20s. In the positive group, the probability of belonging to the negative group was twice as high as that of belonging to the neutral group, while in the 30–34-year-old male neutral group, lower educational levels were associated with a higher probability. Men over the age of 25 who were not economically active were more likely to remain in or belong to the negative group than the positive group.

Subjects who considered their lives to be more unsatisfactory and unhealthier had a higher probability of belonging to the neutral or negative groups than the positive group with respect to marriage intentions. More subjects in the negative group led to a higher odds ratio than the neutral group. In the case of subjective satisfaction, in particular, it was significant as age increased. By contrast, the degree of subjective stress was not significant for any age or group.

For groups considering their family relationships to be normal or poor rather than good, the probability of belonging to the neutral or negative groups was significantly higher than that of belonging to the positive group. The positive group was more likely to belong to the negative group than to the neutral group, and the higher the age, the higher the odds ratio. People who considered their family relationships to be poor were more likely to belong to the neutral or negative groups than those who regarded their family relationships as normal. Men aged 20–24 and 25–29 did not differ according to the number of family members with respect to marriage intentions. Men aged 30–34 with two and three family members were less likely to belong to the neutral group than those with one family member. In addition, men aged 30–34 with two family members were less likely than men with one family member to belong to the negative group.

In 2010, the probability of belonging to the neutral and negative marriage intention groups for all ages was higher than the probability of belonging to the positive group. Since 2014, all groups aged 20–24, 25–29, and 30–34 show significantly increased probability of belonging to the neutral group rather than the positive group. In particular, the odds ratio continued to rise by year, peaking in 2018. No significant difference was observed between 2010 and 2012. For the three family member group, the likelihood of belonging to the negative group rather than the positive group increased significantly from 2018 for the 20–24 age group, from 2016 for the group aged 25–29, and from 2018 for the group aged 30–34. The odds ratio for belonging to the negative group was higher than that for belonging to the neutral group and was particularly significant in 2020.

Table 3 presents the multinomial logistic regression analysis results for unmarried women aged 20–34 based on the determinants of positive marriage intentions. A higher final education level in women aged 20–24 than in women aged 25–29 and 30–34 was associated with a higher probability of belonging to the neutral or negative group. For the group with an educational attainment level below high school, the higher the educational background, the more significant the probability that they would belong to the neutral group. The probability of belonging to the negative group was also significant for those whose final educational attainment level was graduate school.

Positive marriage intentions for groups with a household income of 2 million won or less were associated with no significant differences among women aged 30–34 years. Women between the ages of 20 and 24 with a household income of 4–6 million won were more likely to have positive marriage intentions than women with a household income of 2 million won or less. For women aged 25 to 29, a household income of 6 million won or more increased the likelihood that they would have positive rather than neutral marriage intentions.

Compared to those who were satisfied with their daily life, more dissatisfied subjects showed a higher probability of belonging to the neutral or negative marriage intention groups. In addition, those with poorer subjective satisfaction were twice as likely to belong to the negative group than to the neutral group. Women who considered themselves unhealthy were more likely to have neutral or negative than positive intentions. The results were more significant for subjects aged 25–29 and 30–34 than for those aged 20–24 years. Subjective stress level was not associated with any differences.

Those who considered their relationships with family members to be normal or poor were more likely to have neutral or negative than positive marriage intentions. In particular, those aged 30–34 who considered their family relationships to be poor had a strong probability of belonging to the negative group. Women aged 20–24 and 25–29 showed no difference in marriage values according to number of family members. A significant difference emerged in women aged 30–34, where women living with other family members were more likely to be positive than women living alone.

Women’s marriage intentions changed according to age across the survey years. As of 2010, for women aged 20–24 and 25–29 years the probability of having a neutral rather than a positive position from 2014 onwards increased significantly with each period. From 2016, women aged 20–24 and 25–29 were significantly more likely to have negative rather than positive intentions, showing particularly high probability in 2020. Women aged 30–31 were more likely to have positive than negative marriage intentions in 2012 when compared to 2010 but were more likely to have negative marriage intentions rather than positive in 2018.

## 4. Discussion

Given that the prevailing tendency for Korea’s young adults to marry late or to opt out of marriage entirely has been identified as a key cause of the extremely low fertility rate, and that this tendency is intensifying, it is necessary to understand the changes in marriage intentions and values among this younger generation. The determination of whether marriage intention impacts marriage according to the degree of change in associated values may form an important basis for appreciating the overall flow of society in relation to the declining fertility rates [3,42]. Therefore, this study assessed the changes in the marriage intentions of unmarried youth using social survey data over the past decade and classified young adults according to gender to identify the factors influencing marriage intentions and values.

This study found that the positive marriage intentions of unmarried men and women aged 20–34 were shifting toward neutrality, but the increase in the proportion of negative marriage intentions was not significant. Meanwhile, the proportion of neutral attitudes was found to have increased significantly, and the decline in positive intentions appears to have shifted toward neutrality rather than negativity. Moreover, examination of factors that influence marriage intention according to gender revealed that negative thoughts about one’s economic activity, the extent of one’s subjective satisfaction with one’s daily life and health, and one’s relationship with family members influenced both women’s and men’s probability of having neutral and negative intentions. The implications of these results are detailed below.

Over time, young adults who had positive intentions with regard to marriage were shifting toward neutral intentions and since 2016, marriage intention scores have been negative in lower age groups. Although the youth marriage intention score has not changed significantly, the score’s steady increase indicates that young adults’ need for marriage is weakening. Accordingly, the number of young adults deciding to marry late or to opt out of marriage is impacted by changes in the younger generation’s values. The findings reflect a situation wherein the attraction of marriage as an institution in Korea is declining overall. Neutral intentions toward marriage have the potential to become positive at any time. However, Korea’s competitive social atmosphere has caused young adults to prioritize immediate survival over thoughts of a future, which may include marriage. This social atmosphere makes it difficult for young adults to implement a traditional life process. Korea’s young adults want to be recognized as autonomous beings, alongside their desire to belong to a group as members of society [44]. To resolve social problems around issues such as marriage and childbirth among Korea’s youth and shift neutral marriage intentions toward positivity, it is necessary to foster a social atmosphere that recognizes the new autonomous values of young adults and acknowledges the importance of both belonging and diversity among young adults. If the younger generation’s autonomous values are respected in an environment that fosters stability, their marriage intentions are likely to shift in a positive direction. To this end, it is necessary to implement forms of psychological and emotional support that can relieve the burden on Korea’s youth and ameliorate its contributing factors.

Men who were not economically active showed neutral or negative marriage intentions, while women showed a positive trend when in their late youth (i.e., in their 30s). For both unmarried men and women, the more negative their subjective evaluation, the higher the tendency to espouse neutral or negative marriage intentions. In general, the higher the quality of employment (i.e., wages and employment status) the higher the probability of marriage among young adults [22]. In particular, economic activity emerged as a key factor in determining intentions to transition to marriage among the older youth. These results highlight the importance of economic factors in men’s attitudes toward marriage and demonstrate that gender norms pertaining to the burden of marriage cost and the responsibility to provide economic support for a family after marriage remain firmly in place. Conversely, women’s economic activities had a negative effect. This suggests a synergy between two key aspects: economic power enables women to choose independence, and economic constraints lead to more active decisions to marry.

Regarding the subjects’ subjective evaluation, those who expressed negative views regarding their overall satisfaction and health were also negative about marriage. This evokes the image of a young man who prioritizes his personal values and survival over contributing to community life. In cases where family relationships were normal or poor, the greater possibility of having neutral or negative marriage intentions highlighted the importance of strong family relationships. Unmarried men and women whose family relationships were characterized by greater emotional stability had higher positive marriage intentions [11] and greater sympathy with one’s family was associated with a higher likelihood of positive marriage intentions [20]. Moreover, as unmarried women valued relationships with family more than married women, they expressed a more positive view of marriage [8,29]. In this study, family relationships were identified as a major factor influencing marriage intention. These findings suggest that positive family values lead to the formation of positive marital values.

The limitations of this study are as follows: First, the social survey data were derived from a longitudinal study conducted nationwide every year, making it possible to track social changes by year. However, since the survey is not a panel survey, it is difficult to track changes during certain times for particular individuals. Second, the data’s limitations made it impossible to determine whether decisions to forego marriage were voluntary or involuntary owing to environmental factors. Third, age, period, and cohort have a linear relationship based on a single point and have complementary characteristics that cannot be separated from one another. It is necessary to identify the individual effects of each factor by applying age–period–cohort (APC) analysis to the changes in young adults and their marriage intentions. However, APC analysis was not applied in this study because it only yields clear results for data spanning more than 40 years [45].

## 5. Conclusions

This study is meaningful in that it illuminates the changing attitudes toward marriage among Korea’s youth. The increase in neutral and negative intentions toward marriage in the younger generation indicates that social obstacles make it difficult for young adults to marry. To resolve this, society should create a foothold for the implementation of marriage rather than simply recognizing changing values along with the necessity of marriage. In present-day society, where survival is the goal, it is not easy to marry based only on the desire to do so. Nonetheless, most younger adults remain positive about marriage. A better appreciation of younger adults’ values could be facilitated by the establishment of a dedicated department for youth affairs. It is also necessary to foster a social and economic environment conducive to marriage through the development of policies that consider all young adults and does not support only the underprivileged.

## Figures and Tables

**Table 1:** Marriage intentions and demographic characteristics of unmarried youth aged 20–34 years.

*n* (%)							
Characteristics		Positive (*n* = 18,664)		Neutral (*n* = 19,035)		Negative (*n* = 1511)	
Age ***	20–24	5410	(29.0)	5735	(30.1)	542	(35.9)
	25–29	6177	(33.1)	5913	(31.1)	503	(33.3)
	30–34	7077	(37.9)	7387	(38.8)	466	(30.8)
Gender ***	Men	10,573	(56.6)	7772	(40.8)	509	(33.7)
	Women	8091	(43.4)	11,263	(59.2)	1002	(66.3)
Area *	Metropolitan	5086	(27.3)	5369	(28.2)	450	(29.8)
	Non-metropolitan	13,578	(72.7)	13,666	(71.8)	1061	(70.2)
Final educational ***	Under high school	3596	(19.3)	3902	(20.5)	358	(23.7)
	University (2–3 years)	5527	(29.6)	5796	(30.4)	440	(29.1)
	University (4 years)	8603	(46.1)	8558	(45.0)	667	(44.1)
	Graduate school	938	(5.0)	779	(4.1)	46	(3.0)
Economic activity ***	Yes	11,475	(61.5)	10,909	(57.3)	825	(54.6)
	No	7189	(38.5)	8126	(42.7)	686	(45.4)
Household income ** (Won)	Less than 2 million	5063	(27.1)	5254	(27.6)	461	(30.5)
	2 to 4 million	8276	(44.3)	8131	(42.7)	610	(40.4)
	4 to 6 million	3519	(18.9)	3693	(19.4)	282	(18.7)
	6 million or more	1806	(9.7)	1957	(10.3)	158	(10.5)
Subjective satisfaction ***	Satisfaction	16,548	(88.7)	16,121	(84.7)	1061	(70.2)
	Unsatisfactory	2116	(11.3)	2914	(15.3)	450	(29.8)
Health evaluation ***	Healthy	12,693	(68.0)	11,306	(59.4)	721	(47.7)
	Unhealthy	5971	(28.3)	7729	(35.0)	790	(40.8)
Subjective stress **	Good	17,379	(3.6)	17,885	(5.6)	1397	(11.4)
	Bad	1285	(93.1)	1150	(94.0)	114	(92.5)
Family relationships ***	Good	13,374	(6.9)	11,663	(6.0)	731	(7.5)
	Normal	4900	(71.7)	6715	(61.3)	635	(48.4)
	Bad	390	(26.3)	657	(35.3)	145	(42.0)
Number of family members **	One	2386	(2.1)	2518	(3.5)	227	(9.6)
	Two	3435	(12.8)	3347	(13.2)	227	(15.0)
	Three	5786	(18.4)	5773	(17.6)	466	(15.0)
	Four or more	7057	(31.0)	7397	(30.3)	591	(30.8)
Year ***	2010	3946	(37.8)	2555	(38.9)	211	(39.1)
	2012	3872	(21.1)	2819	(13.4)	139	(14.0)
	2014	3356	(20.7)	3169	(14.8)	152	(9.2)
	2016	2843	(18.0)	3442	(16.6)	279	(10.1)
	2018	2283	(15.2)	3756	(18.1)	296	(18.5)
	2020	2364	(12.2)	3294	(19.7)	434	(19.6)

* *p* < 0.05; ** *p* < 0.01; *** *p* < 0.001 (Bonferroni-corrected *p*-value).

**Table 2:** Factors affecting marriage intentions of unmarried men (aged 20–34) ^1^.

	OR (S.E.)											
	20–24 (*n* = 5479)				25–29 (*n* = 6186)				30–34 (*n* = 7189)			
	Neutral		Negative		Neutral		Negative		Neutral		Negative	
**Demographic characteristics**												
Area (metropolitan = 1)												
Non-metropolitan	−0.329	(0.07)	0.719	(0.18)	0.998	(0.06)	0.973	(0.17)	0.952	(0.06)	1.191	(0.19)
Final educational level (Under high school = 1)												
University (2–3 years)	0.341	(0.38)	1.407	(1.05)	1.385	(0.15) *	0.983	(0.39)	1.542	(0.12) ***	4.606	(0.61) *
University (4 years)	−0.242	(0.38)	0.785	(1.05)	1.304	(0.15)	0.793	(0.38)	1.456	(0.11) ***	4.014	(0.60) *
Graduate school	0.020	(0.38)	1.020	(1.04)	1.192	(0.14)	0.852	(0.37)	1.281	(0.11) *	2.499	(0.60)
**Economic characteristics**												
Economic activity (yes = 1)												
No	0.259	(0.06)	1.296	(0.18)	1.367	(0.06) ***	1.811	(0.16) ***	1.362	(0.07) ***	1.655	(0.21) *
Household income (Won) (less than 2 million = 1)												
2 to 4 million	0.238	(0.08)	1.269	(0.23)	1.054	(0.07)	1.010	(0.19)	0.801	(0.07) ***	1.000	(0.20)
4 to 6 million	0.382	(0.10)	1.466	(0.27)	1.077	(0.09)	0.946	(0.26)	0.838	(0.08) *	0.912	(0.28)
6 million or more	−0.108	(0.11)	0.898	(0.36)	0.879	(0.11)	0.844	(0.31)	0.988	(0.11)	0.750	(0.43)
**Subjective evaluation characteristics**												
Subjective satisfaction (Satisfaction = 1)												
Unsatisfactory	0.523	(0.09)	1.687	(0.22) *	1.407	(0.08) ***	2.341	(0.19) ***	1.220	(0.07) **	1.814	(0.20) **
Health evaluation (Healthy = 1)												
Unhealthy	1.029	(0.07) ***	2.799	(0.17) ***	1.376	(0.06) ***	1.997	(0.16) ***	1.265	(0.05) ***	1.456	(0.17) *
Subjective stress (good = 1)												
Bad	0.355	(0.09)	1.425	(0.24)	0.769	(0.11) *	0.759	(0.29)	0.765	(0.12) *	0.921	(0.37)
**Family characteristics**												
Family relationships (Good = 1)												
Normal	0.660	(0.07) ***	1.934	(0.18) ***	1.489	(0.06) ***	1.427	(0.17) *	1.657	(0.06) ***	2.281	(0.18) ***
Bad	1.115	(0.18) *	3.049	(0.36) **	1.631	(0.17) **	3.118	(0.32) ***	1.735	(0.18) **	7.297	(0.32) ***
Number of family members (One = 1)												
Two	−0.179	(0.11)	0.836	(0.35)	0.919	(0.09)	0.867	(0.25)	0.824	(0.08) *	0.566	(0.27) *
Three	0.207	(0.10)	1.230	(0.29)	0.930	(0.09)	0.884	(0.23)	0.827	(0.08) *	0.784	(0.23)
Four or more	−0.137	(0.10)	0.872	(0.30)	0.955	(0.09)	0.757	(0.25)	0.857	(0.08)	0.748	(0.25)
**Year**												
2012	−0.337	(0.11)	0.714	(0.38)	1.081	(0.10)	0.554	(0.33)	1.161	(0.09)	0.691	(0.30)
2014	0.125	(0.11) ***	1.133	(0.35)	1.920	(0.10) ***	0.830	(0.33)	1.741	(0.09) ***	1.041	(0.29)
2016	0.633	(0.11) ***	1.884	(0.33)	2.603	(0.10) ***	2.295	(0.26) **	2.056	(0.09) ***	1.632	(0.27)
2018	1.238	(0.11) ***	3.449	(0.31) ***	3.497	(0.10) ***	2.607	(0.27) ***	2.719	(0.09) ***	2.012	(0.28) *
2020	1.209	(0.11) ***	3.352	(0.31) ***	2.880	(0.10) ***	4.035	(0.25) ***	2.552	(0.10) ***	3.014	(0.28) ***
χ^2^	472.11 ***				562.17 ***				548.47 ***			
Log-likelihood	−4131.4				−4630.7				−5282.9			

^1^ Reference group = positive intentions for marriage; * *p* < 0.05; ** *p* < 0.01; *** *p* < 0.001; OR, odds ratio; S.E., standard error.

**Table 3:** Factors affecting marriage intentions of unmarried women (aged 20–34) ^1^.

	OR (S.E.)											
	20–24 (*n* = 6208)				25–29 (*n* = 6407)				30–34 (*n* = 7741)			
	Neutral		Negative		Neutral		Negative		Neutral		Negative	
**Demographic characteristics**												
Area (metropolitan = 1)												
Non-metropolitan	0.921	(0.06)	0.831	(0.13)	1.016	(0.06)	0.914	(0.13)	0.988	(0.05)	1.011	(0.14)
Final educational level (Under high school = 1)												
University (2–3 years)	1.800	(0.22) **	2.838	(0.63)	0.973	(0.12)	1.351	(0.31)	1.035	(0.11)	1.585	(0.31)
University (4 years)	1.990	(0.22) **	3.121	(0.62)	1.067	(0.12)	1.121	(0.30)	1.063	(0.11)	1.534	(0.30)
Graduate school	2.129	(0.21) ***	3.951	(0.61) *	1.056	(0.11)	1.159	(0.29)	1.082	(0.10)	1.195	(0.30)
**Economic characteristics**												
Economic activity (yes = 1)												
No	1.084	(0.06)	1.075	(0.12)	1.087	(0.06)	1.188	(0.13)	0.924	(0.05)	0.653	(0.13) **
Household income (Won) (less than 2 million = 1)												
2 to 4 million	0.898	(0.08)	0.778	(0.16)	0.907	(0.07)	0.823	(0.16)	0.893	(0.07)	0.732	(0.16)
4 to 6 million	0.792	(0.09) **	0.625	(0.19) *	0.942	(0.09)	0.824	(0.20)	0.923	(0.08)	0.890	(0.20)
6 million or more	0.929	(0.11)	0.821	(0.21)	0.773	(0.11) *	0.711	(0.24)	1.016	(0.11)	1.212	(0.26)
**Subjective evaluation characteristics**												
Subjective satisfaction (Satisfaction = 1)												
Unsatisfactory	1.222	(0.09) *	2.382	(0.15) ***	1.389	(0.08) ***	3.549	(0.15) ***	1.424	(0.08) ***	3.074	(0.15) ***
Health evaluation (Healthy = 1)												
Unhealthy	1.125	(0.06) *	1.608	(0.12) ***	1.306	(0.06) ***	1.608	(0.13) ***	1.437	(0.05) ***	1.615	(0.13) ***
Subjective stress (good = 1)												
Bad	1.028	(0.12)	1.273	(0.22)	0.780	(0.12) *	0.637	(0.29)	0.684	(0.13) **	1.104	(0.32)
**Family characteristics**												
Family relationships (Good = 1)												
Normal	1.300	(0.06) ***	1.796	(0.13) ***	1.339	(0.06) ***	1.700	(0.14) ***	1.492	(0.05) ***	2.785	(0.14) ***
Bad	1.548	(0.16) **	2.465	(0.25) ***	1.431	(0.16) *	3.309	(0.25) ***	1.876	(0.17) ***	8.056	(0.26) ***
Number of family members (One = 1)												
Two	1.156	(0.12)	0.898	(0.25)	0.917	(0.10)	0.946	(0.23)	0.770	(0.11) *	0.446	(0.25) **
Three	1.143	(0.11)	1.002	(0.22)	0.977	(0.10)	0.991	(0.22)	0.761	(0.11) *	0.583	(0.23) *
Four or more	1.097	(0.11)	1.121	(0.21)	1.057	(0.10)	0.939	(0.22)	0.707	(0.11) **	0.496	(0.23) **
**Year**												
2012	1.161	(0.09)	0.859	(0.27)	1.146	(0.09)	0.821	(0.25)	1.082	(0.08)	0.514	(0.23) **
2014	1.365	(0.09) ***	1.069	(0.27)	1.472	(0.09) ***	1.007	(0.26)	1.399	(0.08) ***	0.869	(0.22)
2016	1.879	(0.10) ***	3.274	(0.23) ***	1.819	(0.09) ***	2.757	(0.22) ***	1.677	(0.08) ***	1.447	(0.20)
2018	3.135	(0.10) ***	5.728	(0.23) ***	3.103	(0.10) ***	4.026	(0.23) ***	2.072	(0.09) ***	1.959	(0.21) **
2020	3.022	(0.11) ***	8.669	(0.23) ***	2.766	(0.10) ***	7.939	(0.21) ***	1.837	(0.09) ***	2.457	(0.21) ***
χ^2^	514.41 ***				569.91 ***				554.61 ***			
Log-likelihood	−5113.3				−5163.4				−6023.1			

^1^ Reference group = positive intentions for marriage; * *p* < 0.05; ** *p* < 0.01; *** *p* < 0.001; ^2^ OR, odds ratio; S.E., standard error.

## Data Availability

The data that support the findings of this study are openly available in “Microdata Integrated Service” in Statistics Korea at https://doi.org/10.23333/P.101018.001, accessed on 20 April 2021, reference number 16.
